# Bio-functionalized nickel-silica nanoparticles suppress bacterial leaf blight disease in rice (*Oryza sativa* L.)

**DOI:** 10.3389/fpls.2023.1216782

**Published:** 2023-08-02

**Authors:** Yasmine Abdallah, Yasser Nehela, Solabomi Olaitan Ogunyemi, Munazza Ijaz, Temoor Ahmed, Ranya Elashmony, Dalal Hussien M. Alkhalifah, Wael N. Hozzein, Lihui Xu, Chengqi Yan, Jianping Chen, Bin Li

**Affiliations:** ^1^ State Key Laboratory of Rice Biology and Breeding, Ministry of Agriculture Key Laboratory of Molecular Biology of Crop Pathogens and Insects, Key Laboratory of Biology of Crop Pathogens and Insects of Zhejiang Province, Institute of Biotechnology, Zhejiang University, Hangzhou, China; ^2^ Department of Plant Pathology, Faculty of Agriculture, Minia University, ElMinya, Egypt; ^3^ Department of Agricultural Botany, Faculty of Agriculture, Tanta University, Tanta, Egypt; ^4^ Department of Biology, College of Science, Princess Nourah bint Abdulrahman University, Riyadh, Saudi Arabia; ^5^ Botany and Microbiology Department, Faculty of Science, Beni-Suef University, Beni-Suef, Egypt; ^6^ Institute of Eco-Environmental Protection, Shanghai Academy of Agricultural Sciences, Shanghai, China; ^7^ Institute of Biotechnology, Ningbo Academy of Agricultural Sciences, Ningbo, China; ^8^ State Key Laboratory for Managing Biotic and Chemical Threats to the Quality and Safety of Agro-products, Key Laboratory of Biotechnology in Plant Protection of Ministry of Agriculture and Zhejiang Province, Institute of Plant Virology, Ningbo University, Ningbo, China

**Keywords:** biosynthesis, nanoparticle composites, rice bacterial leaf blight, *Xanthomonas oryzae* pv. *oryzae*, biofilm

## Abstract

**Introduction:**

Bacterial leaf blight (BLB) caused by *Xanthomonas oryzae* pv*. oryzae* (*Xoo*) is one of the most devastative diseases that threatens rice plants worldwide. Biosynthesized nanoparticle (NP) composite compounds have attracted attention as environmentally safe materials that possess antibacterial activity that could be used in managing plant diseases.

**Methods:**

During this study, a nanocomposite of two important elements, nickel and silicon, was biosynthesized using extraction of saffron stigmas (*Crocus sativus* L.). Characterization of obtained nickel-silicon dioxide (Ni-SiO_2_) nanocomposite was investigated using Fourier transform infrared spectroscopy (FTIR), X-ray diffraction (XRD), Transmission/Scanning electron microscopy (TEM/SEM), and energy-dispersive spectrum (EDS). Antibacterial activities of the biosynthesized Ni-SiO_2_ nanocomposite against *Xoo* were tested by measuring bacterial growth, biofilm formation, and dead *Xoo* cells.

**Results and discussions:**

The bacterial growth (OD_600_) and biofilm formation (OD_570_) of *Xoo* treated with distilled water (control) was found to be 1.21 and 1.11, respectively. Treatment with Ni-SiO_2_ NPs composite, respectively, reduced the growth and biofilm formation by 89.07% and 80.40% at 200 μg/ml. The impact of obtained Ni-SiO_2_ nanocomposite at a concentration of 200 μg/ml was assayed on infected rice plants. Treatment of rice seedlings with Ni-SiO_2_ NPs composite only had a plant height of 64.8 cm while seedlings treated with distilled water reached a height of 45.20 cm. Notably, *Xoo*-infected seedlings treated with Ni-SiO_2_ NPs composite had a plant height of 57.10 cm. Furthermore, Ni-SiO_2_ NPs composite sprayed on inoculated seedlings had a decrease in disease leaf area from 43.83% in non-treated infected seedlings to 13.06% in treated seedlings. The FTIR spectra of biosynthesized Ni-SiO_2_ nanocomposite using saffron stigma extract showed different bands at 3,406, 1,643, 1,103, 600, and 470 cm^−1^. No impurities were found in the synthesized composite. Spherically shaped NPs were observed by using TEM and SEM. EDS revealed that Ni-SiO_2_ nanoparticles (NPs) have 13.26% Ni, 29.62% Si, and 57.11% O. *Xoo* treated with 200 µg/ml of Ni-SiO_2_ NPs composite drastically increased the apoptosis of bacterial cells to 99.61% in comparison with 2.23% recorded for the control.

**Conclusions:**

The application of Ni-SiO_2_ NPs significantly improved the vitality of rice plants and reduced the severity of BLB.

## 1 Introduction

Rice (*Oryza sativa* L.) is the most consumed cereal crop worldwide. Food and Agriculture Organization considers rice as an important crop for food security in the world (Wang et al., 2023). One of the most serious diseases infecting rice plants is bacterial leaf blight (BLB) caused by *Xanthomonas oryzae* pv. *oryzae* (*Xoo*). BLB is a dominant diseases among various rice varieties (Singh et al., 2015). Infection by *Xoo* reduces the efficiency of photosynthesis and metabolism of rice plants, which subsequently leads to yield loss of up to 80% (Yasmin et al., 2017; Ma et al., 2023).

Diverse management strategies have been applied to control plant diseases. Use of chemical bactericides could be effective in controlling BLB. However, because of the extensive application of traditional chemical bactericides and antibiotics, it may catalyze mutations and lead to durable resistant races of pathogenic bacteria (Russo et al., 2008; Xu et al., 2010).

Marques et al. (2009) reported that the widespread occurrence of copper and streptomycin resistance in field isolates and its adaptation to bactericides have a negative impact on the chemical management of *Xanthomonas campestris* pv. *viticola*. In China, different studies have reported streptomycin resistance in various phytopathogens. Choi et al. (2015) concluded that more than 50% of tested field strains of *Pseudomonas syringae* pv. *tabaci* showed medium- to high-level resistance to streptomycin. The evolution of *Xoo* strains in overcoming single-gene–based resistance has been reported. For instance, Xa4, a single-based breeding gene for BLB management has been defeated by *Xoo* sub-population evolution (Shanti et al., 2010).

Use of nanoparticles (NPs) to combat plant diseases is one of the best tools to enhance pathogen suppression while maintaining an eco-friendly and safe method as it results in the bioreduction of metals to stable metallic NPs through a green route (Melo et al., 2018). While many NPs have existed, currently, they have not been widely applied in plant pathology. However, the recent use of nano-medicine against human pathogens has re-evolution plant disease management approach (Elmer et al., 2018). Recently, NPs of metallic oxides (single and composites) have gained momentum in phytopathology. The antibacterial action of NPs against phytopathogens is confirmed by many studies (Abdallah et al., 2020). For instance, ZnO NPs are found to be efficient against different pathogenic bacteria including *Xoo* and fungi (Ogunyemi et al., 2019). In addition, Cai et al. (2018) reported the antibacterial action of magnesium oxide NPs against *Ralstonia solanacearum*. The physicochemical characteristics of NPs increase their interaction with bacteria and improves their anti-microbial activities (Aziz et al., 2016; Rudramurthy et al., 2016; Ihtisham et al., 2021). NPs bind to the pathogen’s cell wall causing deformation of cell membranes due to high-energy transfer and, subsequently, lead to the death of the pathogen (Pereira et al., 2022). In bacteria, metal NPs (MNPs) increase cell membrane permeability and cell destruction. Among known NPs, nickel has gained wide interest as an antifungal and antibacterial element. Nickel-based NPs have been used for controlling several plant pathogenic fungi (Ahmed et al., 2016; Markowicz, 2023). Jeyaraj Pandian et al. (2016) and Mirhosseini et al. (2018) reported a high growth inhibition against Gram-negative bacteria and *Candida* species (*C. albicans* and *C. tropicalis*) by using nickel oxide (NiO) NPs.

To improve NP properties and increase their efficacy, the synthesis of nanocomposites was recently tested for that purpose (Prakasham et al., 2010; Baig et al., 2021). Omanović-Mikličanin et al. (2020) explained that the synthesis of nanocomposites consists of an assemblage of two different natural materials, which introduces material with greater performance characteristics than that of the original components separately. Lattuada and Hatton (2011) and Tao et al. (2008) reported that nanocomposites include the interaction between their different materials, and these interactive nanocomposites usually possess distinct properties that are not expressed in their individual elemental components. One of the most popular inert support materials is silica (Liou, 2004; Adam and Andas, 2007). The potential changes in the characterization of NiO NPs and their efficacy as a bactericide, when combined with supported component such as silica, and the role it could play in management of BLB are not known. Therefore, this study will investigate the potential use of such a nanocomposite in suppressing *Xoo* resulting in decreased disease severity.

MNPs are synthesized by many physiochemical methods such as co-precipitation, sol-gel, microemulsion, hydrothermal reaction, electrospray synthesis, and laser ablation. Biogenic methods such as using plant extracts can also be used for the synthesis of MNPs. Biosynthesis of NPs via plant extracts is economical, eco-friendly, and non-hazardous (Dubey et al., 2010; Alharbi et al., 2022). The ability of various plants (pomegranate, rose, banana, hibiscus, geranium leaves, cinnamomum, aloe, and basil) for the synthesis of NPs has been studied (Sathishkumar et al., 2009; Ahmad et al., 2010; Philip, 2010). Saffron (*Crocus sativus*), a bulbous perennial belonging to the iris family (*Iridaceae*) (Siddiqui et al., 2018), has been successfully used in the biosynthesis of several MNPs (Abootorabi et al., 2016; Bagherzade et al., 2017). The aqueous extract of saffron stigmas has OH groups from variety of phenolic compounds. These OH groups improve saffron’s capability for the biosynthesis of NPs, as it reacts with metal ions and plays a role for the reduction of metal raw materials to MNPs (Khan and Rizvi, 2014). Therefore, this study aims to biosynthesize nickel-silicon NP composite using extract of saffron stigmas, to characterize the obtained composite, and to investigate the antibacterial action of obtained biosynthesized nickel and silica NPs against *Xoo* and its impact on rice plants challenged with *Xoo*.

## 2 Materials and methods

### 2.1 Extraction of aqueous saffron

Aqueous saffron extraction was carried out in accordance to the method of Amin et al. (2017). A gram of dried saffron stigmas was added to 100 ml of deionized water in a beaker and then placed in a water bath for 4 h at 60°C. The extract was filtered twice using filter paper Whatman no.1 that was used directly for the synthesis of Ni-SiO_2_ NP composite.

### 2.2 Biosynthesis of Ni-SiO_2_ NP composite

To synthesize Ni-SiO_2_ composite, a 100-ml solution of each element (1 mM bulk NiO and 1 mM bulk SiO_2_) was prepared separately by adding previously prepared 100 ml of aqueous saffron extract to each one and then stirred at 180 Revolution Per Minute (rpm) for 4 h at 60°C. Then, Ni-SiO_2_ composite was prepared by mixing the previously prepared solutions of NiO and SiO_2_ using a ratio of 1:1 (v/v). The new mixture was swirled for 4 h at 60°C. The final solution was divided into 50-ml tubes and centrifuged (10,000 rpm/20 min). The pellets were retrievd and washed gently using ddH_2_O. Obtained pellets were lyophilized for 8 h.

### 2.3 Characterization of Ni-SiO_2_ composite

To evaluate the formation of Ni-SiO2 NPs in the obtained powder, Fourier transform infrared spectroscopy (FTIR) analysis was done by employing spectrometer (Vector 22, Bruker, Germany) at the range of 500–4,000 cm^−1^ region at a resolution of 4 cm^−1^. X-ray diffraction (XRD) was adopted to test the purity of obtained particles, and the mean crystallite size from XRD was calculated adopting the Scherrer equation (Jeffery, 1957). Transmission electron microscopy (TEM) was employed to observe morphology of NPs using (JEM-1230, JEOL, Akishima, Japan). Obtained NP powder was scanned by scanning electron microscopy (SEM) using (TM-1000, Hitachi, Japan). The SEM microscope was connected to energy-dispersive spectrum (EDS) to be assured of the presence of the elements.

### 2.4 *In vitro* inhibitory effect of Ni-SiO_2_ NP composite and determination of minimum inhibition of concentration

*Xoo* strain GZ 0005 used for this investigation was collected from the Institute of Biotechnology, College of Agriculture and Biotechnology, Zhejiang University, China. The virulence of *Xoo* was tested and confirmed before the study. The antibacterial activity of Ni-SiO_2_ NP composite against *Xoo* was evaluated by using the agar well diffusion assay as explained by Monteiro et al. (2013). An overnight 100 µl of *Xoo* culture (approximately 1 × 10^8^ Colony forming unit (CFU)/ml) was added to 5 ml of Nutrient Agar (NA) medium, and, then, 50 µl each of previously prepared concentration (Ni-SiO_2_ NP composite at 50, 100, and 200 μg/ml) was poured into 6-mm-diameter agar wells. Five replications were done for this assay; each replication was typified by a plate consisting of a well for each of the three concentrations. The plates were incubated at 30°C for 48 h. The clearance zone around the well was scaled after 48 h. The experiment was repeated following the same condition.

The minimum inhibition of concentration (MIC) of Ni-SiO_2_ NP composite against *Xoo* was investigated as explained by Wiegand et al. (2008). In detail, 100 µl of an overnight culture of *Xoo* (approximately 1 × 10^8^ CFU/ml) was poured into sterile tubes containing 5 ml of nutrient broth. Ni-SiO_2_ NP composite was added to each tube, and the concentrations were adjusted to 50, 100, and 200 µg/ml each in respective tube. The tubes were kept in 30°C with shaking at approximately 180 rpm. After 48 h of incubation, MIC was measured using a UV spectrophotometer by the optical density at 600 nm (OD_600_). The investigation was repeated twice.

### 2.5 Effect of Ni-SiO_2_ NP composite on biofilm formation of *Xoo*

The ability of Ni-SiO_2_ NP composite to inhibit *Xoo* biofilm was measured as described by Merritt et al. (2005). A 100 μl of overnight *Xoo* culture (1 × 10^8^ CFU/ml) was added to Nutrient Broth (NB) medium containing Ni-SiO_2_ NP composite to get a final concentration of 50, 100, and 200 μg/ml. The mixture was kept static in a 30°C incubator for 48 h to develop a biofilm in a 96-well plate. To stain the attached biofilm, crystal violet (CV) was added to the wells after discarding the supernatant. CH_3_COOH (33%) was used in solubilizing the CV attached to the biofilm and measured at OD_570_.

### 2.6 Live/dead assays to infer the cell membrane integrity

Fluorescence emitted from propidium iodide (PI) of dead bacterial cells after incubation with the NPs was measured by flow cytometer (Kumar et al., 2011). *Xoo* culture (1 × 10^8^ CFU/ml) was centrifuged (5,000 rpm/5 min), and Ni-SiO2 NP (200 µg/ml) composite was added to the obtained pellets for 4 h. PI was added in the dark for 30 min to stain the chromatin of bacterial cells. Subsequently, the dead cell ratio of *Xoo* cells was measured by flow cytometry (FC) (Gallios Beckman Coulter, Germany).

### 2.7 Effect of Ni-SiO_2_ composite on rice seedlings infected with *Xoo*

The experiment was conducted as complete randomized blocks. Five replications were used per treatment. Three rice seedlings (cv. II You 023 *Oryza sativa* L.) in each replicate were sown in small pots filled with sterile soil and kept in the growth chamber under 28 ± 2°C, 80% relative humidity with a photoperiod of 16-h light and 8-h dark. This experiment consisted of four treatments that include the following:

1. In the first treatment, 3-week-old rice seedlings were sprayed with a suspension of Ni-SiO_2_ NP composite (200 µg/ml); after 48 h, the rice seedlings were inoculated with *Xoo* strain GZ 0005 culture (1 × 10^8^ CFU/ml) via leaf clipping.
2. In the second test treatment, 3-week-old rice seedlings were sprayed with distilled water; after 48 h, the rice seedlings were inoculated with *Xoo* strain GZ 0005 culture (1 × 10^8^ CFU/ml) via leaf clipping.
3. In the third test treatment, 3-week-old rice seedlings were sprayed with a suspension of Ni-SiO_2_ NP composite (200 µg/ml), and no *Xoo* inoculation was applied.
4. The fourth treatment, 3-week-old rice seedlings were sprayed with distilled water, and no *Xoo* inoculation was applied.

The experiment was carried out at 11:00 a.m. to ensure that the stomata had opened. Diseased leaf area, plant height, and fresh and dry biomass weight were recorded 1 month after application of Ni-SiO_2_ NP composite on rice plants. The percentage of diseased leaf area (DLA%) was calculated as follows:


DLA%=Total lesion area of the test sample/Total leaf area of the test sample×100%

### 2.8 Statistical analysis

Data were subjected to analysis of variance using SAS, 2003 software (SAS Institute, Cary, NC, USA). The general linear model procedure was used to check the significant differences among the main treatments. Individual comparisons between mean values were performed using Duncan’s method (*P* ≤ 0.05). Simple linear regression (SLR) analysis was performed to better understand the relationship between concentrations of Ni-SiO_2_ NP composite and inhibition zone, bacterial growth inhibition, and biofilm formation inhibition. The fitted regression model was stated as a regression equation, coefficient of determination (R^2^), ${\text{R}}_{\text{adj}}^{2}$ , and p-value as determined by the F-test (*P* ≤ 0.05).

## 3 Results

### 3.1 Characterization of Ni-Si O_2_ NP composite

**Figure 1:**
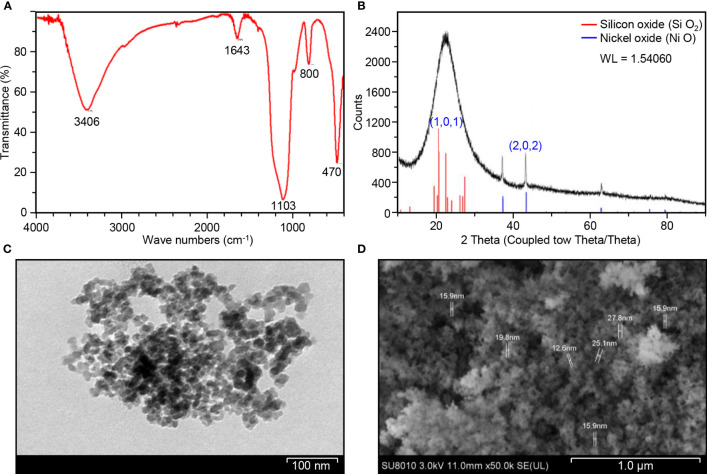
Structural and compositional characterization of Ni-SiO_2_ NP composite. **(A)** FTIR spectrum of Ni-SiO_2_ NPs. **(B)** X-ray diffraction patterns of Ni-SiO_2_ NP composite. **(C)** Bright-field TEM image of Ni-SiO_2_ NP composite. **(D)** SEM image of Ni-SiO_2_ NP composite.

**Figure 2:**
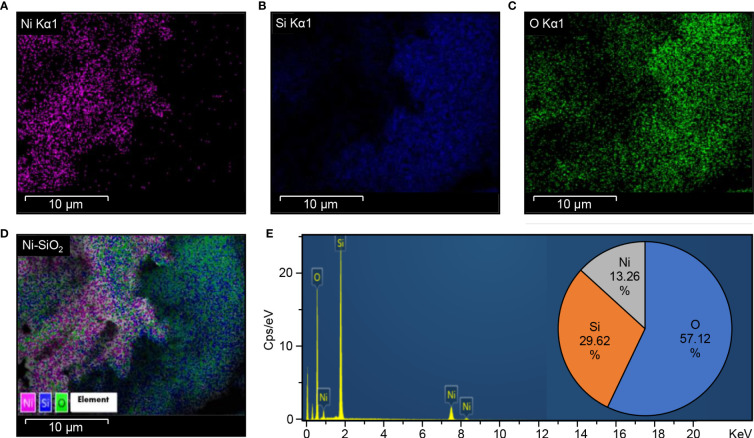
Energy dispersion spectrum (EDS) Ni-SiO_2_ NP composite. **(A)** Ni Kα1, **(B)** Si Kα1, **(C)** O Kα1, **(D)** Ni-SiO_2_, and **(E)** composite. Energy-dispersive spectrum showing the predominance of Ni, Si, and O elements and the percentage of each element in the Ni-SiO_2_ NP composite.

The FTIR spectra of biosynthesized Ni-SiO_2_ NP composite revealed various bands at 3,406, 1,643, 1,103, 800, and 470 cm^−1^ (**Figure 1A**). The band at 3,406 cm^−1^ was assigned to hydroxl stretch hydrogen bonds, the band at 1,643 cm^−1^ was related to C=C stretch, and the band at 1,103 cm^−1^ indicates C–O stretches. The peaks at 800 and 470 cm^−1^ were attributed to the symmetric vibration of Si atoms. XRD pattern showed no impurities in tested samples. The sharpest diffraction peaks were recorded at 2θ around 43°, which can be indexed as (202) for nickel, and at 2θ around 20°, representing (101) for silica (**Figure 1B**). Spherically shaped NPs were observed by using TEM (**Figure 1C**) and SEM (**Figure 1D**). Data from the EDS of Ni (**Figure 2A**), Si (**Figure 2B**), O (**Figure 2C**), and Ni-SiO_2_ NPs (**Figure 2D**) revealed that Ni-SiO_2_ NP composite has 13.26% Ni, 29.62% Si, and 57.11% O (**Figure 2E**).

### 3.2 Composition of biosynthesized Ni and SiO_2_ NPs enhances their antibacterial activity against *Xoo*

**Figure 3:**
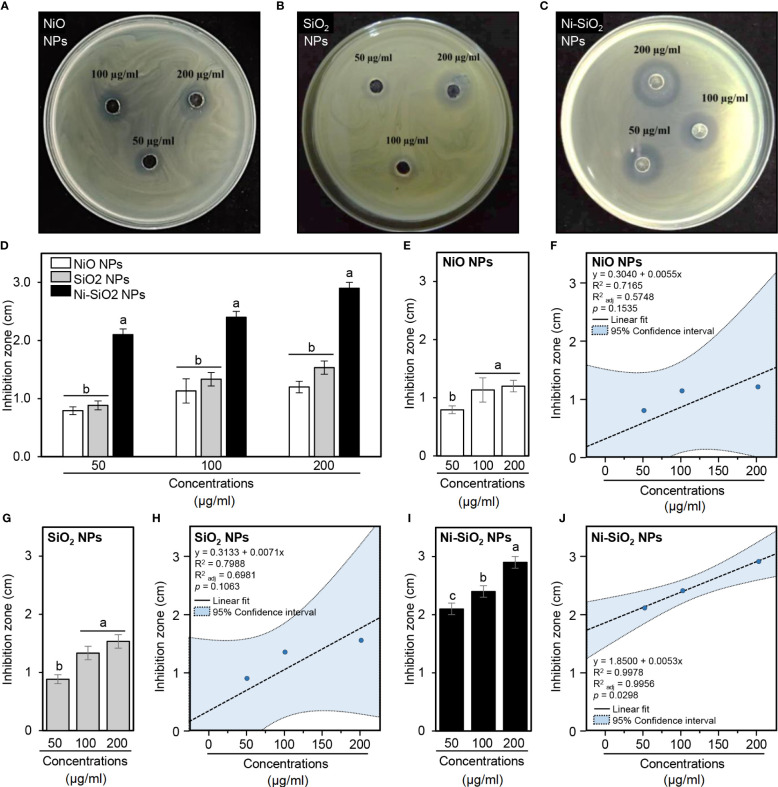
*In vitro* antibacterial action of NiO, SiO_2_, and Ni-SiO_2_ NP composite against *Xoo*. **(A–C)** Antibacterial activity of different concentrations (50, 100, and 200 µg/ml) NiO, SiO_2_, and Ni-SiO_2_ NP composite, respectively, against *Xoo*. **(D)** Diameters of the inhibition zones of *Xoo* after the treatment of NPs (50, 100, and 200 µg/ml). **(E, G, I)** Diameters of the inhibition zones of *Xoo* after the treatment with different concentrations of NiO, SiO_2_, and Ni-SiO_2_ NP composite, respectively. Vertical bars represent the means ± standard deviation (means ± SD) of three biological replicates (n = 3). Different letters indicate statistically significant differences among treatments, whereas bars followed by the same letter(s) are not significantly different (*P* ≤ 0.05). **(F, H, J)** Simple linear regression between concentrations (µg/ml) NiO, SiO_2_, and Ni-SiO_2_ NP composite, respectively, and the inhibition zones (cm). The linear fit regression line is presented as a dashed line, whereas the 95% confidence intervals are light blue–shaded and edged by dotted lines. Regression equations, R2, R2_adj_, and p-value based on the F-test (*P<* 0.05) were also obtained and presented within the graph.

The ability of Ni, Si, and Ni-SiO_2_ NPs to inhibit *Xoo* bacteria was investigated by using plate assay technique (**Figures 3A–C**, respectively). Three concentrations (50, 100, and 200 µg/ml) of each NP were tested. In general, all the tested NPs had a dose-dependent antibacterial action against *Xoo* that significantly inhibited its growth *in vitro* (**Figure 3D**). It is worth mentioning that the Ni-SiO_2_ NP composite was the most efficient NPs suppressing *Xoo* growth (**Figures 3C, D**). The three tested concentrations of Ni-SiO_2_ NP composite (50, 100, and 200 µg/ml) produced inhibition zones of 2.1, 2.4, and 2.9 cm, respectively, compared with 0.9, 1.3, and 1.5 cm for SiO_2_ and 0.8, 1.1, and 1.2 cm for NiO NPs (**Figure 3D**). The MIC of the Ni-SiO_2_ NPs was 200 μg/ml, in which *Xoo* growth was inhibited by 89.07%, whereas using 50 and 100 μg/ml resulted in 21.50% and 54.37% inhibition, respectively.

In general, *in vitro* experiments showed that NiO NPs efficiently suppressed the bacterial growth of *Xoo* in a concentration-dependent fashion with no significant differences between the two highest concentrations (100 and 200 µg/ml) (**Figure 3E**). However, SLR between NiO NP concentrations (µg/ml) and inhibition zone (cm) showed a positive correlation between them (y = 0.3040 + 0.0055x, R2 = 0.7165, R2_adj_ = 0.5748, and *P* = 0.1535; **Figure 3F**). Likewise, the antibacterial activity of SiO_2_ NPs was identical to that of NiO NPs (**Figure 3G**) even without significant differences between them at all studied concentrations (**Figure 3D**). Moreover, SLR showed a positive correlation between the concentrations of SiO_2_ NPs and clearance zone (y = 0.3133 + 0.0071x, R2 = 0.7988, R2_adj_ = 0.6981, and *P* = 0.1063; **Figure 3H**). Furthermore, the most effective NP, Ni-SiO_2_ composite, exhibited a clear progressive increase in inhibition zones (**Figure 3I**), which was strongly correlated with its concentrations (y = 1.8500 + 0.0053x, R2 = 0.9978, R2_adj_ = 0.9956, and *P* = 0.0298; **Figure 3J**).

### 3.3 Ni-SiO_2_ NP composite inhibits bacterial growth of *Xoo* in nutrient broth

**Figure 4:**
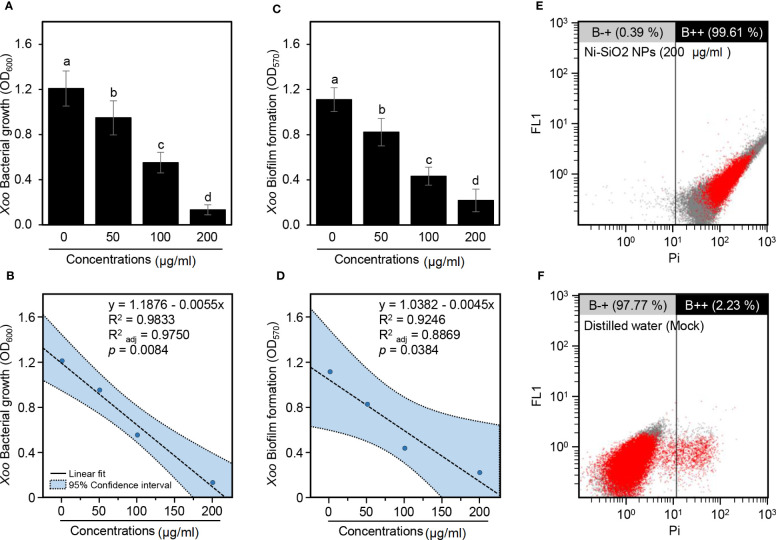
Antibacterial activity of Ni-SiO_2_ NP composite against *Xoo*. **(A)** Bacterial growth of *Xoo* in nutrient broth containing different concentrations of Ni-SiO_2_ NP composite (0, 50, 100, or 200 µg/ml) as indicated by optical density at 600 nm (OD_600_). **(B)** Simple linear regression between concentrations of Ni-SiO_2_ NP composite (µg/ml) and *Xoo* Bacterial growth (OD_600_). **(C)** Biofilm formation of *Xoo* after the treatment with different concentrations of Ni-SiO_2_ NP composite (0, 50, 100, or 200 µg ml^−1^) as indicated by optical density at 570 nm (OD_570_). **(D)** Simple linear regression between concentrations of Ni-SiO_2_ NP composite (µg/ml) and *Xoo* biofilm formation (OD_570_). **(E, F)** Flow cytometry observations of *Xoo* cells after incubation with Ni-SiO_2_ NP composite (200 μg/ml) or distilled water, respectively. In panels **(A)** and **(C)**, bars represent the means ± standard deviation (means ± SD) of three biological replicates (n = 3). Different letters indicate statistically significant differences among treatments (*P* ≤ 0.05). In panels **(B)** and **(D)**, the linear fit regression line is presented as a dashed line, whereas the 95% confidence intervals are light blue–shaded and edged by dotted lines). Regression equations, R2, R2_adj_, and P-value based on the F-test (P< 0.05) were also obtained and presented within the graph.

Furthermore, because of the superiority of Ni-SiO_2_ NP composite over NiO and SiO_2_ NPs, the focus was placed on it throughout the rest of this study. Briefly, in nutrient broth, Ni-SiO_2_ NP composite significantly inhibited the growth of *Xoo* in a dose-dependent manner as revealed by OD_600_ (**Figure 4A**). In other words, the inhibition extents increased from 50< 100< 200 µg/ml. In agreement with these findings, SLR showed strong negative correlation (y = 1.1876 − 0.0055x, R^2 = ^0.9833, ${\text{R}}_{\text{adj}}^{2}$ = 0.9750, and *P* = 0.0084) between *Xoo* bacterial growth (OD_600_) and Ni-SiO_2_ NP concentrations (µg/ml) (**Figure 4B**).

### 3.4 Ni-SiO_2_ NP composite inhibits biofilm formation of *Xoo*

Likewise, Ni-SiO_2_ NP composite significantly hindered biofilm development of *Xoo* cells in a dose-dependent manner because the higher concentrations showed lower biofilm formation, and vice versa, as indicated by optical density at 570 nm (OD_570_) (**Figure 4C**). Antibiofilm activity of 25.91%, 61.06%, and 80.40% were detected as a result of using Ni-SiO_2_ NPs of 50, 100, and 200 µg/ml, respectively. In addition, SLR showed a strong negative correlation (y = 1.0382 − 0.0045x, R^2 = ^0.9246, ${\text{R}}_{\text{adj}}^{2}$ = 0.8869, and *P* = 0.0384) between biofilm formation (OD_570_) and Ni-SiO_2_ concentrations (µg/ml) (**Figure 4D**).

### 3.5 Ni-SiO_2_ NP composite causes cell injury or death to *Xoo*

Moreover, cell damage/apoptosis of *Xoo* cells was assessed using FC and PI-based method. Briefly, incubation of *Xoo* with Ni-SiO_2_ NP composite (200 µg/ml) drastically increased the apoptosis of the bacterial cells to 99.61% (**Figure 4E**) compared with 2.23% for the mock control (distilled water; **Figure 4F**). Together, in addition to the inhibition of bacterial growth, our findings proved that Ni-SiO_2_ NP composite might cause cell puncture or death to *Xoo* when it was amended with a concentration of 200 µg/ml.

### 3.6 Application of Ni-SiO_2_ NP composite improves plant growth and reduces disease severity of BLB in rice

**Figure 5:**
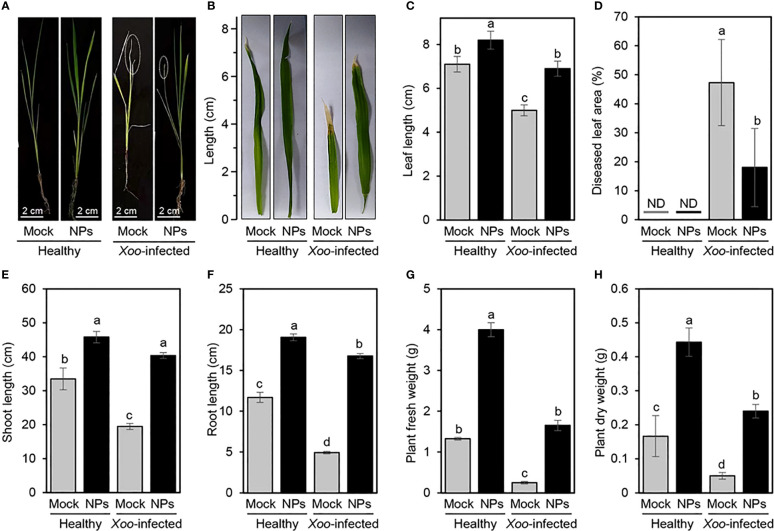
Effect of application of Ni-SiO_2_ NP composite on rice growth and disease severity of bacterial blight disease of rice caused by *Xanthomonas oryzae* pv. *oryzae* **(A, B)** Ni-SiO_2_-treated vs. non-treated healthy and *Xoo*-infected rice plants and leaves, respectively. **(C)** Leaf length (cm), **(D)** diseased leaf area (%), **(E)** shoot length (cm), **(F)** root length (cm), **(G)** plant fresh weight (g), and **(H)** plant dry weight (g) of Ni-SiO_2_treated vs. non-treated healthy and *Xoo*-infected rice plants. Bars represent the means ± standard deviation (means ± SD) of three biological replicates (n = 3). Different letters indicate statistically significant differences among treatments (*P* ≤ 0.05). Mock = Control.

A notable improvement in rice growth was observed when Ni-SiO_2_ NP composite was applied at a concentration of 200 µg/ml as a foliar application on healthy and *Xoo*-infected rice plants under greenhouse conditions (**Figure 5A**). Interestingly, Ni-SiO_2_ NP composite application notably increased the leaf length of treated rice plants (**Figures 5B, C**); however, it reduced the total diseased leaf area in *Xoo*-infected rice plants (**Figure 5D**). Accordingly, the disease leaf area decreased from 43.83% in non-treated control plants to 13.06% when Ni-SiO_2_ NP composite was applied to infected plants (**Figure 5D**). Moreover, the amendment with Ni-SiO_2_ NPs significantly increased the height of non-infected rice plants to 64.8 cm in comparison with 45.2 cm of plants amended with only water (**Figure 5E**). Likewise, treating *Xoo*-infected rice plants with Ni-SiO_2_ NP composite significantly increased plant height to 57.1 cm compared with non-treated infected rice plants, which appeared short with an average plant height just below 20 cm. Similarly, the application of Ni-SiO_2_ NPs produced almost the same pattern in terms of root length (**Figure 5F**). In addition to improving rice growth, using Ni-SiO_2_ NP composite showed a positive effect on biomass. Briefly, application of Ni-SiO_2_ NPs significantly increased both fresh (**Figure 5G**) and dry (**Figure 5H**) weight of treated healthy and *Xoo*-infected rice plants compared with non-treated ones.

## 4 Discussion

NPs have been applied in the field of agriculture as highly effective bactericides, fungicides, and nano fertilizers due to their small size, large surface area, and high reaction (Elmer and White, 2018; Hossain et al., 2019; Ogunyemi et al., 2019). The synthesis of NPs produced a variety of morphology, sizes, and compositions that were determined by numerous physical, chemical, and biological techniques (Pagar et al., 2023). Our study aimed to biosynthesize Ni-SiO_2_ NP composite with new properties that could contribute to the management of BLB by using extraction of saffron stigmas (*Crocus sativus* L.).

Studies of the infrared spectrum were conducted to explore the potential mechanism behind the formation of Ni-SiO_2_ NP composite and information about the functional groups (Irshad et al., 2018; Petousis et al., 2020). The FTIR spectra of biosynthesized Ni-SiO_2_ NP composite revealed various peaks that confirmed the presence of important bonds such as hydroxyl stretch, C=C stretch, C–H, and Si–O–Si bond (Majewski et al., 2013; Adel et al., 2022). Upon reviewing infrared spectrum results, plant extract of saffron stigmas could be responsible for the bio-reduction of Ni-SiO_2_ NP composite. Moreover, silica bonds contributed to the stability of NiO NPs. Phytochemicals easily show the ability to synthesize nickel NPs (Singh et al., 2016; Shwetha et al., 2021). The obtained results show that there are no impurities revealed by the XRD pattern in biosynthesized Ni-SiO_2_ composite.

Silica was able to improve the morphological characteristics of nickel NPs including particles size. Spherically shaped NPs were observed by using TEM and SEM. The size of obtained nickel-silica composite averaged between 12.6 and 27.8 nm. Our finding matches a study by Saha et al. (2015), which was able to synthesize Ni NPs of a size range of 10–30 nm in Ni-SiO_2_ composite prepared by sol-gel route. The size of the produced Ni NPs was smaller in comparison with that of other synthesis protocol (Lajevardi et al., 2013).

Data collected from EDS revealed that Ni-Si-O NP composite has 13.26% Ni, 29.62% Si, and 57.11% O. As reported by Saha et al. (2015), one Si atom reacts with two O_2_ atoms to form SiO_2_. Thus, 29.62% Si present in the composite combines with 57.11% O_2_ to produce SiO_2_. The crystalline nature of synthesized Ni-SiO_2_ NP composite was investigated by XRD technique. The wide spectrum range of 20° and 30° is attributed to the presence of an amorphous Si matrix. The formation of NiO is exempted from the phase analysis by XRD.

Ni-SiO_2_ NP composite was able to inhibit *Xoo* growth and significantly increase the ratio of *Xoo* dead cells to 99.61% compared with 2.23% for control. Therefore, according to the obtained results, Ni-SiO_2_ NP composite can be used as bactericides that have antimicrobial activity as documented by Ahmed et al. (2016) and Jeyaraj Pandian et al. (2016). As NPs have positive or low negative charges, they are electrostatically attracted and adhered to the negatively charged cell membrane of bacteria (Zein El-Abdeen and Farroh, 2019). Subsequently, it caused irregular pit formations on the cell wall of the pathogenic bacteria that facilitate the entry of NPs into periplasmic space and inside bacterial cells (Ninganagouda et al., 2014; Wang et al., 2023). The high efficacy of Ni-SiO_2_ NP composite against *Xoo* could be due to the size of the nickel NPs that have been reduced by silicon to range approximately from 10 to 30 nm, which allows nickel NPs to intensively enter the bacterial cell, resulting in ion accumulation that contributes to membrane porosity damaging the cytoplasm and cell structures. This destruction of cell structure caused the escape of the embedded cell contents, leading to bacterial cell death (Jeyaraj Pandian et al., 2016; Zhu et al., 2022).

The inhibition of biofilm formation, which was detected by using Ni-SiO_2_ NP composite, confirms and matches that of the previous studies on metal oxide NPs (Lee et al., 2014). Le Ouay and Stellacci (2015) and Pellieux et al. (2000) documented that the inhibitory effect of NPs on bacteria is linked to the formation of Reactive Oxygen Species (ROS). ROS promotes oxidative stress in cells and induces DNA, protein, lipids, and cell damage (Piao et al., 2011; Zhang et al., 2018). In addition to the bactericidal effect of Ni-SiO_2_ NP composite, it enhanced rice growth and significantly increased the height of the plant. It also showed a positive effect on rice seedlings’ biomass fresh and dry weight. Mirzajani et al. (2014) and Syu et al. (2014) reported that rice treated with NPs enhanced root growth, which may be due to the interaction between NPs and ROS scavenging, hormone signaling pathways, and auxin. Tarafdar et al. (2014) and Zafar et al. (2016) stated that metal oxide NPs shows enhancement on shoot length of *Pennisetum americanum* and *Brassica nigra*.

This study proved that the application of Ni-SiO_2_ NP composite significantly decreased the biofilm of *Xoo*, which subsequently decreased the virulence of the bacteria. The treatment with MgO and MnO_2_ NPs at the primary stages of growth caused a promotion in rice seedlings growth and increased the photosynthetic parameters while reducing BLB expression (Ogunyemi et al., 2023). On the basis of this report, it can be inferred that, because NPs had a positive impact on photosynthesis, the plant yield will invariably be positively affected. Xu et al. (2021) reported that the application of titanium dioxide NPs on two different cultivars of rice (WYJ23 and YY2640) significantly increased the agronomic data and yield. Therefore, on the basis of reports of the positive impacts of NPs application, it indicates that the treatment of rice with NPs improves both the agronomic trait and yield of rice irrespective of the cultivar or NPs used.

This present work provides helpful and useful insights for using the Ni-SiO_2_ NP composite as potent applications for antibacterial activities. Ni-SiO_2_ NP composite, which is cheap, stable, and nontoxic, indicates a promising safe result that can be used not only in the management of plant diseases but also as a medical treatment for human diseases. Ni NPs were used for their antibacterial activity in the field of medicine and were found to be effective when used for targeting cancer cells (Sudhasree et al., 2014; Ezhilarasi et al., 2016). Hence, despite numerous reports about the antibacterial activity of individual NP elements against *Xoo*, there are few studies of the nanocomposites against this pathogen (Namburi et al., 2021; Chauhan et al., 2023). Therefore, the report of this study is novel, which helps to bridge the gap of the management of *Xoo* using Ni-SiO_2_ NP composite.

## 5 Conclusion

In conclusion, the use of saffron stigma extract in biosynthesizing Ni-SiO_2_ NPs successfully produced a pure composite. The composite of nickel-silica particles have a small size range of 12.6–27.8 nm. The composite had the ability to inhibit *Xoo* growth to the point where 89.07% of *Xoo* cells were killed when treated with Ni-SiO_2_ NP composite (200 µg/ml). The obtained composite also showed that the bacterial anti-biofilm activity reached 80.40% and achieved 99.61% dead cells of *Xoo.* The application of Ni-SiO_2_ NP composite significantly promoted the growth of rice plants challenged with *Xoo* compared with untreated plants. Ni-SiO_2_ NP composite increased biomass fresh and dry weight. In general, Ni-SiO_2_ NP composite is a promising effective tool for suppressing *Xoo* infection on rice plants. On the basis of the potent antibacterial activity of the synthesize nanocomposite recorded in this study, we hereby suggest future studies to be conducted on the mechanism of nanocomposite on ROS and phytohoromones and their effect on rice plants yield using different cultivars.

## Data availability statement

The original contributions presented in the study are included in the article/supplementary material. Further inquiries can be directed to the corresponding authors.

## Author contributions

YA: conceptualization, investigation, formal analysis, and writing (original draft). YN, SO, MI, and TA: investigation, formal analysis, and writing (review and editing). RE, DA and WH: validation and writing (review and editing). LX, CY, JC, and BL: conceptualization, supervision, funding acquisition, and writing (review and editing). All authors contributed to the article and approved the submitted version.
